# Rheumatic Heart Disease in Kerala: A Vanishing Entity? An Echo Doppler Study in 5–15-Years-Old School Children

**DOI:** 10.1155/2015/930790

**Published:** 2015-09-14

**Authors:** Bigesh Nair, Sunitha Viswanathan, A. George Koshy, Prabha Nini Gupta, Namita Nair, Ashok Thakkar

**Affiliations:** ^1^Almadeena Institute of Medical Science, Kottakkal, Malappuram, Kerala 676503, India; ^2^Alappuzha Government Medical College, Alappuzha, Kerala 688005, India; ^3^Trivandrum Government Medical College, Trivandrum, Kerala 695011, India; ^4^Amala Medical College, Thrissur, Kerala 680555, India; ^5^Sahajanand Medical Technologies Pvt. Ltd., Surat, Gujarat 395004, India

## Abstract

*Background.* Early detection of subclinical rheumatic heart disease by use of echocardiography warrants timely implementation of secondary antibiotic prophylaxis and thereby prevents or retards its related complications. *Objectives.* The objective of this epidemiological study was to determine prevalence of RHD by echocardiography using World Heart Federation criteria in randomly selected school children of Trivandrum. *Methods. *This was a population-based cross-sectional screening study carried out in Trivandrum. A total of 2060 school children, 5–15 years, were randomly selected from five government and two private (aided) schools. All enrolled children were screened for RHD according to standard clinical and WHF criteria of echocardiography. *Results. *Echocardiographic examinations confirmed RHD in 5 children out of 146 clinically suspected cases. Thus, clinical prevalence was found to be 2.4 per 1000. According to WHF criteria of echocardiography, 12 children (12/2060) were diagnosed with RHD corresponding to echocardiographic prevalence of 5.83 cases per 1000. As per criteria, 6 children were diagnosed with definite RHD and 6 with borderline RHD. *Conclusions. *The results of the current study demonstrate that echocardiography is more sensitive and feasible in detecting clinically silent RHD. Our study, the largest school survey of south India till date, points towards declining prevalence of RHD (5.83/1000 cases) using WHF criteria in Kerala.

## 1. Introduction

Rheumatic heart disease (RHD), a consequence of valvular damage caused by an exaggerated immune response to group-A streptococcal infection, usually during childhood, still remains unabated in developing countries [1]. It is currently estimated that at least 15.6 million people have clinically recognized RHD with annual mortality rate between 3 and 12.5% [2–5], which accounts for 200000 to 250000 premature deaths [1].

Several population-based surveys of the school children identified high volume of clinically unrecognized cases detected by echocardiographic screening [6–10]. The low sensitivity of cardiac auscultation for the detection of RHD and thereby underestimation of the disease burden have been recognized in these epidemiological studies. The delay in the early detection or nondetection of subclinical disease leads to advanced stages of RHD and places heavy economic burden on the healthcare system [11]. Thus, early detection of subclinical RHD has been emphasized as timely implementation of secondary prevention measure (penicillin administration, cornerstone of acute rheumatic fever and RHD treatment, at regular intervals to avoid further exposure to group-A streptococcal infection that triggers autoimmune response [1]) is the only cost-effective approach. As a result of body of evidence, World Health Organization (WHO) also recommends echocardiographic screening in RHD endemic areas [12]. In 2012, World Heart Federation (WHF) published evidence-based diagnostic criteria for echocardiographic detection of RHD [13].

India contributes to nearly 25% to 50% of the global burden of RHD [12, 14]. However, literature review identified the need of well-conducted echocardiography based prevalence studies which used internationally accepted standard set of diagnostic criteria. Thus, the present study was designed to obtain accurate prevalence data on rheumatic valvular abnormalities in school children using WHF provided echocardiographic criteria.

## 2. Methods

### 2.1. Study Design and Patient Population

This population-based cross-sectional screening study was carried out in Trivandrum between December 2013 and May 2014. All the schools of the Trivandrum district, Kerala, were stratified into government and private (aided) schools. Five government and two private schools were selected randomly. We approached the principals of the schools to participate in the study. After the consent of the principals, all the students (of the selected schools) were invited to participate and were thoroughly informed about the study. The children whose parents or guardian gave written informed consent (in Malayalam, a local language) were included in the study. We performed multistage random sampling. The protocol of the study was approved by institutional ethics committee before the commencement of the study.

### 2.2. Methodology

A total of 2060 school children aged 5–15 years from five government (*n* = 1023 students) and two private (*n* = 1037 students) schools were screened during the study period. Absentees were also noted and revisits were made to examine all of them.

The participating children were interviewed using a proforma. As poverty and overcrowding are the risk factors for the occurrence of the disease, we included socioeconomic status, type of housing (kutcha house), and number of family members of the enrolled children. Houses made from mud, thatch, or other low-quality materials are called katcha houses. Socioeconomic status was determined as per the classification provided by Dudala et al. [15]. For each child, any history of rheumatic fever was noted.

All the enrolled school children underwent clinical examination. Cardiac auscultation was performed with the patients in the supine and left lateral decubitus position. Children in whom an apical systolic murmur was detected clinically underwent echocardiography for the confirmation of RHD. These children were classified as having clinically detected RHD.

WHF criteria of echocardiography were used to identify rheumatic valvular abnormalities. These criteria were designed for use in RHD endemic populations to identify asymptomatic individuals who had no history of acute rheumatic fever. According to the criteria, the children identified with rheumatic valvular abnormalities were diagnosed with either “definite RHD” or “borderline RHD” as follows.

*World Heart Federation Criteria of Echocardiographic Diagnosis of Rheumatic Heart Disease*

*Echocardiographic Criteria for Individuals Aged ≤ 20 Years*

 Definite RHD (either (A), (B), (C), or (D)) are as follows.
  - Pathological MR and at least two morphological features of RHD of the MV.
  - MS mean gradient ≥4 mmHg.
  - Pathological AR and at least two morphological features of RHD of the AV.
  - Borderline disease of both the AV and MV.
 Borderline RHD (either (A), (B), or (C)) are as follows.
  - At least two morphological features of RHD of the MV without pathological MR or MS.
  - Pathological MR.
  - Pathological AR.
 Normal echocardiographic findings (all of (A), (B), (C), and (D)) are as follows.
  - MR that does not meet all four Doppler echocardiographic criteria (physiological MR).
  - AR that does not meet all four Doppler echocardiographic criteria (physiological AR).
  - An isolated morphological feature of RHD of the MV (e.g., valvular thickening) without any associated pathological stenosis or regurgitation.
  - Morphological feature of RHD of the AV (e.g., valvular thickening) without any associated pathological stenosis or regurgitation.

*Criteria for Pathological Regurgitation*

 Pathological MR (all four Doppler echocardiographic criteria must be met) is as follows.
  (i) Seen in two views.
  (ii) In at least one view, jet length ≥2 cm.
  (iii) Velocity ≥3 m/s for one complete envelope.
  (iv) Pansystolic jet in at least one envelope.
 Pathological AR (all four Doppler echocardiographic criteria must be met) is as follows.
  (i) Seen in two views.
  (ii) In at least one view, jet length ≥1 cm.
  (iii) Velocity ≥3 m/s in early diastole.
  (iv) Pandiastolic jet in at least one envelope.

*Morphological Features of RHD*

 Features in the MV are as follows.
  - AMVL thickening ≥3 mm (age-specific).
  - Chordal thickening.
  - Restricted leaflet motion.
  - Excessive leaflet tip motion during systole.
 Features in the AV are as follows.
  - Irregular or focal thickening.
  - Coaptation defect.
  - Restricted leaflet motion.
  - Prolapse.

Definite RHD children were given secondary prophylaxis and asked to follow-up in Trivandrum medical college which is a tertiary hospital. Children with borderline RHD were asked to follow up every six months for an echo to determine progression of disease.

### 2.3. Sample-Size Calculation

Our sample-size calculation was based on the results of previous study of the echocardiographic prevalence of rheumatic heart disease. Bhaya et al. [16] estimated the echocardiographic prevalence of RHD to be 51 per 1,000 (95% confidence interval: 38–64 per 1,000) in 1059 school children aged 6–15 years of Bikaner city, India. According to the following equation, the estimated sample size was 1787. However, we enrolled 2060 school children to estimate echocardiographic prevalence:

(1) $$\begin{array}{c} \text{Sample size}=\frac{4pq}{d^{2}}, \end{array}$$

where *p* = 5.1%, echocardiographic prevalence of RHD being 51 per 1000 (published study); *q* = 100 − *p*, and *d* is 20% of *p*.

### 2.4. Statistical Analysis

Statistical analyses were performed using SPSS Statistics V.15.0. Continuous variables are presented as mean ± SD. Categorical data are expressed as frequency (percentages).

## 3. Results

A total of 2060 school children from five government (*n* = 1023 students) and two private (*n* = 1037 students) schools were screened during the study period. The average age of the study population was 12.6 ± 2.1 years with predominantly male subjects (*n* = 1335; 64.8%). The other demographic characteristics are presented in Table 1.

All the enrolled school children underwent clinical examinations which identified pathological murmur in 184 school children (apical systolic murmur in 45 school children). Out of these 184 school children with pathological murmur, echocardiography confirmed RHD in 5 (11.1%), corresponding to the clinical prevalence of 2.4 cases per 1000 children (95% CI, 1.1 to 4.2).

Echocardiographic screening was performed in all 2060 enrolled school children. Of these 2060 school children, congenital anomalies were identified in 32 (1.58%) school children.  Table 2 shows congenital anomalies identified during echocardiographic screening. The most common anomaly was mitral valve prolapse (*n* = 15) and atrial septal defect (*n* = 8). Thus, the echocardiographic prevalence of congenital heart disease is 7.77 cases per 1000 children (95% CI, 3.3 to 10.3). Echocardiographic screening demonstrated mitral regurgitation and aortic regurgitation in 146 and 97 school children. However, WHF pathological mitral regurgitation and aortic regurgitation were observed in 1 and 5 school children. Mitral valve abnormalities such as anterior mitral valve leaflet thickening ≥ 3 mm, chordal thickening, and excessive mitral valve leaflet tip motion during systole were observed in 56, 39, and 12 school children. Irregular or focal thickening of aortic valve, coaptation defect, or prolapse of aortic valve was observed in 3, 5, and 5 screened school children. According to WHF criteria, RHD was diagnosed in 12 school children of whom 6 had definite RHD and 6 had borderline RHD (Table 3). Echocardiographic prevalence of RHD was found to be 5.83 cases per 1000 children (95% CI, 2.5 to 9.1).

## 4. Discussion

RHD consolidates a spectrum of different stages of clinically silent and clinically manifest valvular degeneration culminating in congestive heart failure, increasing the risk of endocarditis and cerebrovascular events and eventually leading to premature death. It is estimated that approximately 80–85% of children younger than 15 years live in the area where this disease is endemic and the disease is considered as leading cause of cardiac disease in children. As per the systematic analysis carried out by Murray et al., RHD causes highest number of disability adjusted life years (DALYs) among 10–14-year-olds (516.6 per 100000 people, 95% CI 425.3–647.0) [17]. Hence, secondary prophylaxis administration in early detected asymptomatic cases of RHD is considered crucial. Thus, over the past decades, interest has been raised in echocardiographic screening of RHD owing to its sensitivity for the detection of valvular abnormalities.

A joint WHO and National Institute of Health (NIH) working party published consensus case definitions for RHD [18]. However, these definitions do not include full spectrum of morphological features of RHD in addition to lack of evidence-support. As a result of these limitations, some of the epidemiological studies adopt different criteria (to define morphological and functional abnormalities) to estimate prevalence of RHD. In a study Marijon et al. considered morphological features as diagnostic criteria along with WHO criteria [19]. They reported that currently recommended WHO criteria miss up to three quarters of cases of subclinically affected and therefore potentially treatable RHD children. A total of 2170 children were included of 6 to 17 years of age. Their results demonstrated varying subclinical evidence of RHD. At on-site examination, among the 208 children (9.6%) with echocardiographic features of at least minimal valve regurgitation, 18 and 124 children were suspected to have RHD according to WHO and combined criteria, respectively. Thereafter, 17 of the 18 were confirmed to have definite RHD according to WHO criteria (94%), whereas 66 of the 124 cases were considered to have definite RHD by the use of combined criteria (53%). The definite echocardiographic RHD prevalences were thus 7.8 per 1000 (95% CI, 4.6 to 12.5) and 30.4 per 1000 (95% CI, 23.6 to 38.5) for WHO and combined criteria. Thus, the set of criteria by WHO suffers from a substantially lower sensitivity; thus, the combined criteria were better suited for screening of subclinical RHD [19].

Different echocardiographic criteria for the diagnosis of RHD may account for the difference of RHD prevalence and essentially make epidemiological comparisons invalid. So, to close this gap, WHF published the first internationally endorsed evidence-based criteria for echocardiographic detection of RHD in 2012 [13]. These guidelines remove clinical examination from the diagnosis and divide disease into definite and borderline as well as providing subcategories within each for different combinations of diseases (isolated valvular regurgitation, isolated morphological change, etc.). However, it allows rapid identification of the disease in those who do not have history of acute rheumatic fever. In India, no study was performed using these criteria till date. The present study is the first and largest echocardiographic based screening of RHD in school children of Kerala till date which utilized WHF criteria for the diagnosis of RHD in children.

In Kerala, Alleppy showed a clinical prevalence of 2.2 per 1000 school children in 1975 [20]. In 2002–2005, Indian Council of Medical Research (ICMR) estimated clinical prevalence of RHD to be 0.12 per 1000 school children of Cochin which was based upon echocardiographic confirmation of the suspected cases [21]. However, the present study estimated echocardiographic prevalence being 5.83/1000 (including subclinical cases), indicating a decline of rheumatic heart disease in Kerala, even though it may be more in some unidentified pockets. Epidemiological studies (after 2000) in school children which estimated prevalence of RHD in India have been summarized in Table 4. However, in these studies, the incidence was based on echocardiographic confirmation of the clinically suspected cases.

In India, there are few studies carried out which estimated echocardiographic prevalence of RHD in children. Bhaya et al. screened 1059 school children aged 6–15 years from coeducational schools of Bikaner city [16]. The prevalence of RHD was found 51 per 1,000 school children (95% CI: 38 to 64 per 1,000 school children) by using WHO criteria of echocardiography. Similarly, a cross-sectional survey carried out by Saxena et al. among 6270 randomly selected school children (aged 5–15 years) using WHO criteria of echocardiography demonstrated echocardiography prevalence of 20.4 per 1000 school children (95% CI: 16.9 to 23.9 per 1000 school children) [9]. However, the estimated prevalence of clinical RHD of the study (carried out by Saxena et al.) was 0.8 per 1000 school children. In our study also, echocardiographic prevalence of RHD (using WHF criteria) was found to be 5.83 cases per 1000 school children (95% CI, 2.5 to 9.1 per 1000 school children) as compared to prevalence of clinical RHD 2.4 cases per 1000 children (95% CI, 1.1 to 4.2). However, direct comparison of echocardiography prevalence of RHD with earlier studies was inappropriate due to different echocardiographic criteria.

Studies in various countries most often replicate the true epidemiology of RHD, however sometimes being baffled by over- or underdiagnosis. Oversimplification may perhaps impact on the ability to detect rheumatic and other pathologies, such that some rare cases of isolated aortic regurgitation may be missed by using a simplified set of criteria. Mirabel et al. suggested a strategy to be implemented in low-income countries: to focus on cost-efficient policies such as prevention (i.e., the use of penicillin) as cardiac surgery more often becomes unaffordable in such settings [22]. Similarly, in another study, a simple focused cardiac ultrasound (FCU) with pocket-sized devices that could be operated by nonexperts seemed feasible and yielded acceptable sensitivity and specificity for RHD detection when compared with the state-of-the-art approach and thereby opened new perspectives for mass screening for RHD in low-resource settings [23].

Thus, our study demonstrates feasibility echocardiographic screening of RHD in school children using WHF criteria in Indian setting as well as providing echocardiographic prevalence of RHD in school children using WHF criteria in India.

## 5. Conclusions

The results of the current study demonstrate that echocardiography is more sensitive in detecting clinically silent cases of RHD. Thereby, systematic screening with echocardiography reveals higher prevalence of RHD as compared with clinical screening. However, our study, the largest school survey of south India till date, points towards declining prevalence of RHD (5.83/1000 cases) using WHF criteria in Kerala. The study also showed feasibility to carry out echocardiographic screening of RHD using WHF criteria in Indian setting.

## Figures and Tables

**Table 1:** Baseline characteristics of the study population.

Characteristics	*N* = 2060
Age	12.6 ± 2.1
Male, *n* (%)	1335 (64.8%)
Height, mean ± SD	148.5 ± 16.8
Weight, mean ± SD	42.0 ± 13.6
Body mass index, mean ± SD	18.8 ± 4.2
Systolic blood pressure, mean ± SD	102.4 ± 12.1
Diastolic blood pressure, mean ± SD	67.8 ± 9.0
Number of family members, mean ± SD	4.4 ± 1.0
Number of rooms, mean ± SD	2.9 ± 1.0
Kutcha type of housing, *n* (%)	457 (22.2%)
Low socioeconomic status, *n* (%)	1002 (48.6%)

**Table 2:** Congenital cardiac anomalies detected by screening echocardiography.

Congenital anomaly	*n*
Mitral valve prolapsed	15
Atrial septal defect	10
Ventricular septal defect	3
Bicuspid aortic valve	2
Dilated aortic root	1
Double outlet right ventricle	1

**Table 3:** Rheumatic heart disease classified according to World Heart Federation criteria.

RHD category	WHF criteria	*n*
Definite RHD		6

Definite RHD (A)	Pathological MR and at least two morphological features of RHD of the MV	5

Definite RHD (B)	MS mean gradient ≥4 mmHg	0

Definite RHD (C)	Pathological AR and at least two morphological features of RHD of the AV	1

Definite RHD (D)	Borderline disease of both the AV and MV	0

Borderline RHD		6

Borderline RHD (A)	At least two morphological features of RHD of the MV without pathological MR or MS	6

Borderline RHD (B)	Pathological MR	0

Borderline RHD (C)	Pathological AR	0

**Table 4:** Epidemiological studies of rheumatic heart disease in school children of different regions of India.

Study	Publication year	Region	Age	Sample size	Prevalence of RHD (per 1000 children)
Jose and Gomathi [24]	2003	Vellore	6–18 years	229829	0.68
Periwal et al. [25]	2006	Bikaner	5–14 years	3292	0.67
Misra et al. [26]	2007	Gorakhpur	4–18 years	118212	0.5
Negi et al. [27]	2013	Shimla (rural and urban)	5–15 years	15145	0.59
